# A Case of Gastric Metastasis of Renal Cell Carcinoma Resected by Endoscopic Submucosal Dissection After Endoscopic Follow‐up of Morphological Changes

**DOI:** 10.1002/deo2.70257

**Published:** 2025-12-01

**Authors:** Yuichi Fukami, Nagako Nishihira, Kazuki Kawakami, Yuka Hayakawa, Yuki Matsui, Midori Wakiya, Masayoshi Fukuda, Hiromichi Shimizu, Kazuo Ohtsuka, Ryuichi Okamoto

**Affiliations:** ^1^ Department of Gastroenterology Tokyo Kyosai Hospital Tokyo Japan; ^2^ Department of Pathology Tokyo Kyosai Hospital Tokyo Japan; ^3^ Department of Gastroenterology and Hepatology Institute of Science Tokyo Tokyo Japan

**Keywords:** endoscopic submucosal dissection, gastric metastasis, morphological change, renal cell carcinoma, subcentimeter gastric lesion

## Abstract

A 62‐year‐old man underwent partial nephrectomy for right renal cell carcinoma (RCC). Four years later, esophagogastroduodenoscopy revealed gastric mucosal redness with a smooth surface. Two more years later, the red lesion showed a morphological change to a reddish elevation, which prompted a biopsy. Histopathological examination led to the diagnosis of gastric metastasis of RCC. As the tumor resided within the submucosa and no other organ metastasis was found after nephrectomy by that time, endoscopic submucosal dissection (ESD) was performed and successfully resected. No evidence of local recurrence was noted for 7 years after ESD. Topical resection, such as ESD, can be considered an option to treat the gastric metastasis of RCC for which there are no established treatment guidelines. In this case report, we aimed to investigate a rare case of gastric metastasis of RCC, which was monitored endoscopically, showing morphological changes over several years, and was successfully resected by ESD.

## 1 Introduction

Metastasis of renal cell carcinoma (RCC) to other organs can be observed most commonly in the lungs, liver, bones, and brain at the time of diagnosis and during postoperative surveillance. The metastasis of RCC to the gastrointestinal tract is not common, and a few relevant case reports can be found [1]. Although some RCCs progress slowly and metastases can be resected depending on their site, there is no defined treatment strategy for gastrointestinal metastasis of RCC in the current treatment guidelines. Here we report a rare case of gastric metastasis of RCC, which was monitored through esophagogastroduodenoscopy (EGD) showing morphological changes over several years, and was successfully resected by endoscopic submucosal dissection (ESD).

## 2 Case Report

The patient was a 62‐year‐old man who had hypertension and was a Hepatitis B virus carrier. He did not take any medications and did not have a history of smoking. He occasionally consumed alcohol. When he was 56 years old, he underwent a partial nephrectomy of a right RCC at our institution. He was pathologically diagnosed with an RCC; clear cell subtype, measuring 45 mm, which was classified as T1b, N0, M0, stage I, suggesting that no further chemotherapy and surgery were needed. As part of a regular health checkup, an EGD was performed, revealing no obvious gastric lesions. The patient was followed up with periodic blood tests and computed tomography (CT) scans for 4–6 months, and annual EGD. Anemia and gastrointestinal bleeding were not detected in the follow‐up period. Carcinoembryonic antigen, carbohydrate antigen 19‐9, and alpha‐fetoprotein were also within the normal limits.

**FIGURE 1:**
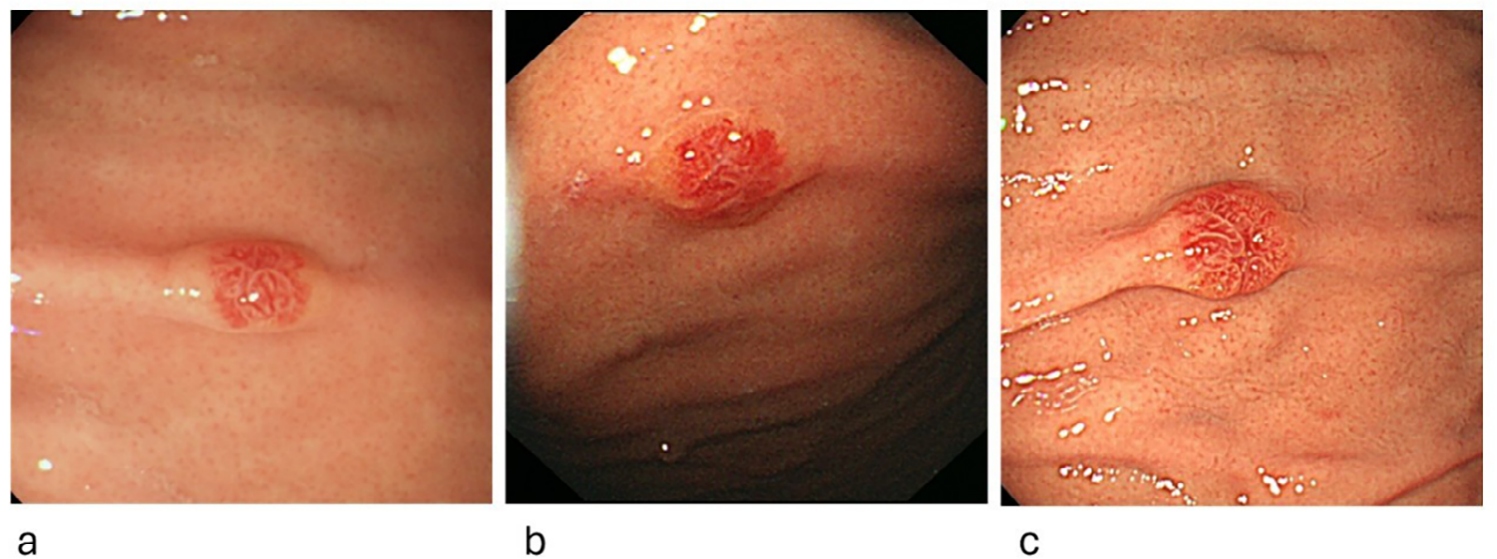
An annual endoscopic observation was performed to monitor the lesion. (a) Four years post‐renal cell carcinoma (post‐RCC) surgery, a 5‐mm area of erythema was noted on the anterior wall of the greater curvature of the gastric body. (b) Five years post‐RCC surgery, the same erythema was observed, with no change in morphology. (c) Six years post‐RCC surgery, a 5‐mm reddish polypoid lesion with central depression was detected at the same site, raising suspicion of a morphological change.

Four years after nephrectomy, EGD revealed a 5‐mm erythematous area on the anterior wall of the greater curvature of the gastric body. No biopsy was performed, and the lesion was monitored (Figure 1a). A year later, EGD showed the same erythema with no obvious change in morphology, and follow‐up was continued (Figure 1b). However, six years after nephrectomy, the EGD results revealed that erythema showed morphological changes to a 5‐mm reddish elevated lesion with central depression at the same site. A biopsy was then performed, and histopathological examination led to the diagnosis of gastric metastasis of RCC (Figure 1c).

**FIGURE 2:**
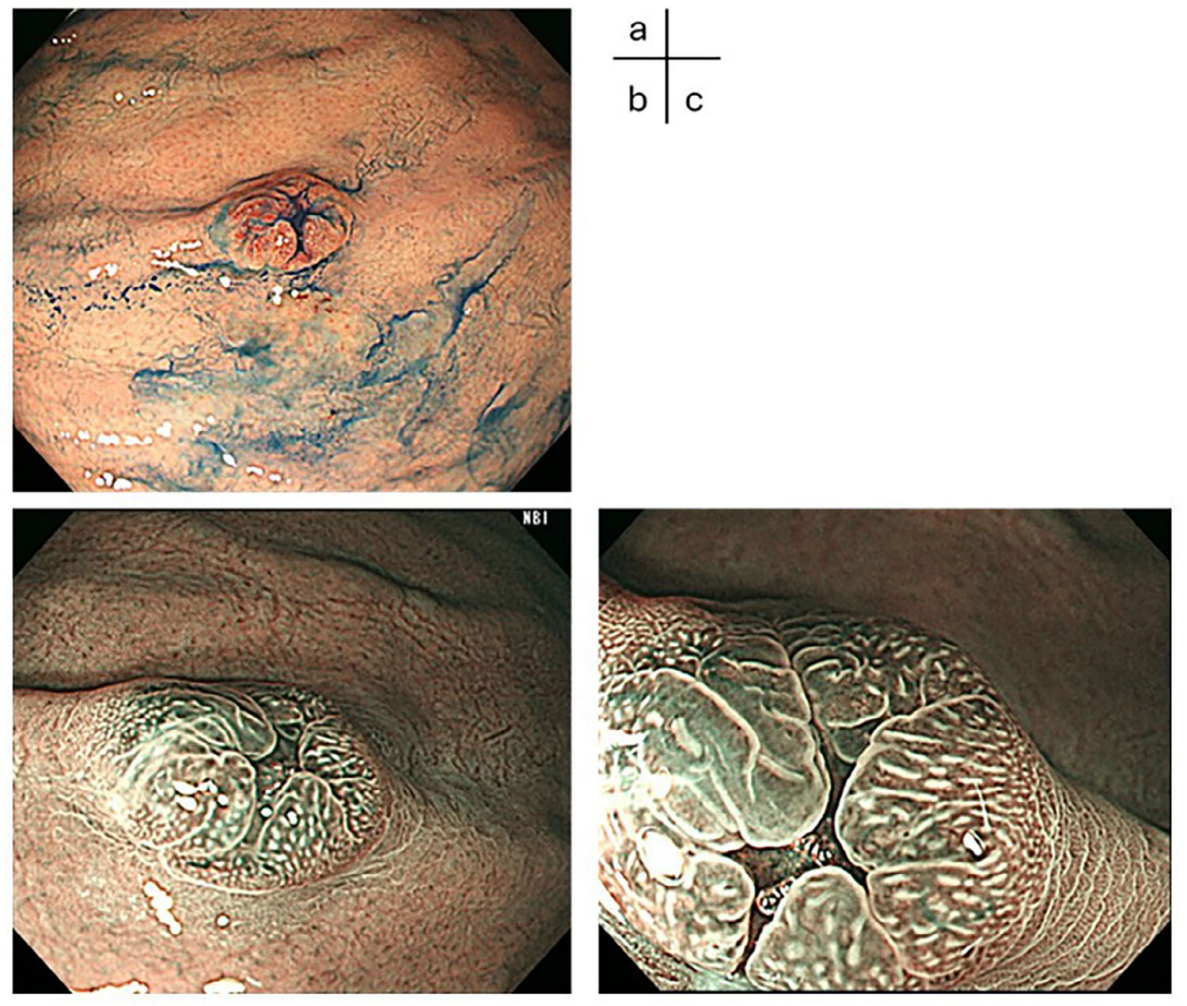
The lesion was examined in indigo carmine dye spraying and narrow‐band imaging (NBI). (a) Indigo carmine dye spraying showing a central depression. (b, c) NBI‐magnifying endoscopy revealed no abnormal vascular structures on the surface.

Indigo carmine dye spraying suggested the presence of a central depression (Figure 2a). Narrow‐band imaging revealed no abnormal vascular structures on the surface (Figure 2b,c).

**FIGURE 3:**
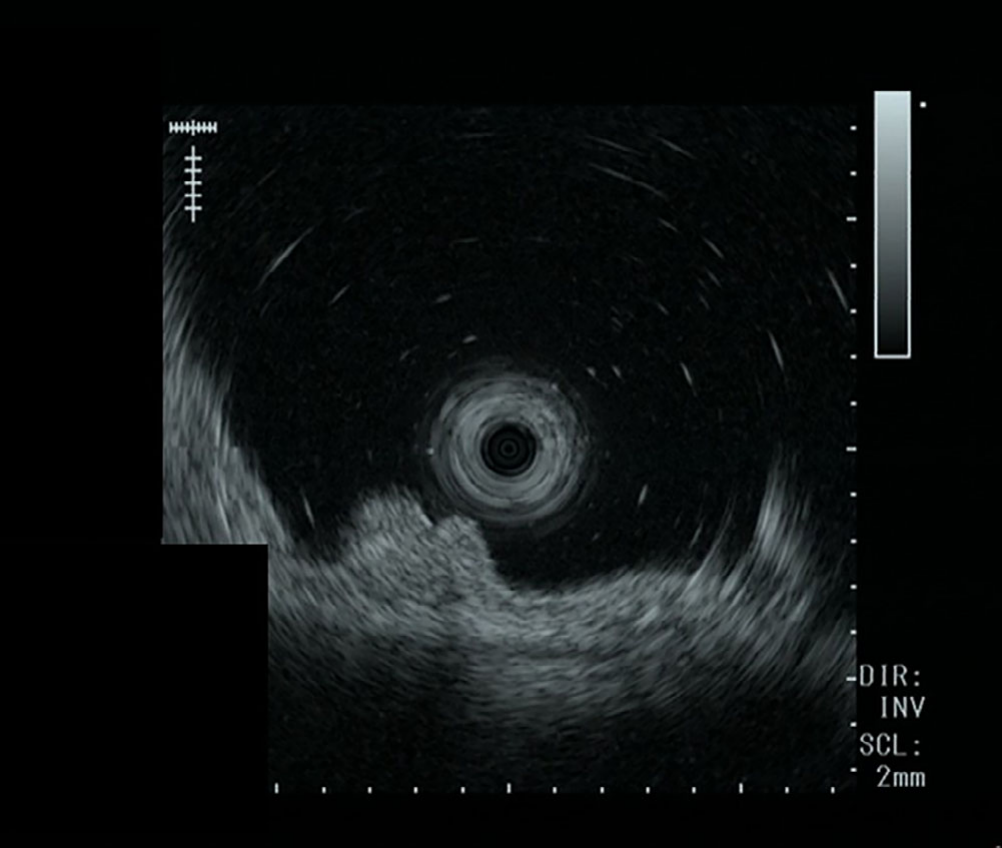
Endoscopic ultrasonography (EUS) image. EUS revealed a 5‐mm hypoechoic tumor within the submucosal layer.

Additional endoscopic ultrasonography revealed a 5‐mm hypoechoic tumor residing in the submucosal layer (Figure 3). Although chemotherapy was considered as one of the therapeutic options, the tumor resided in the submucosa with very slow progression, and no other organ metastasis was found after nephrectomy by that time. ESD was performed after obtaining informed consent from the patient.

**FIGURE 4:**
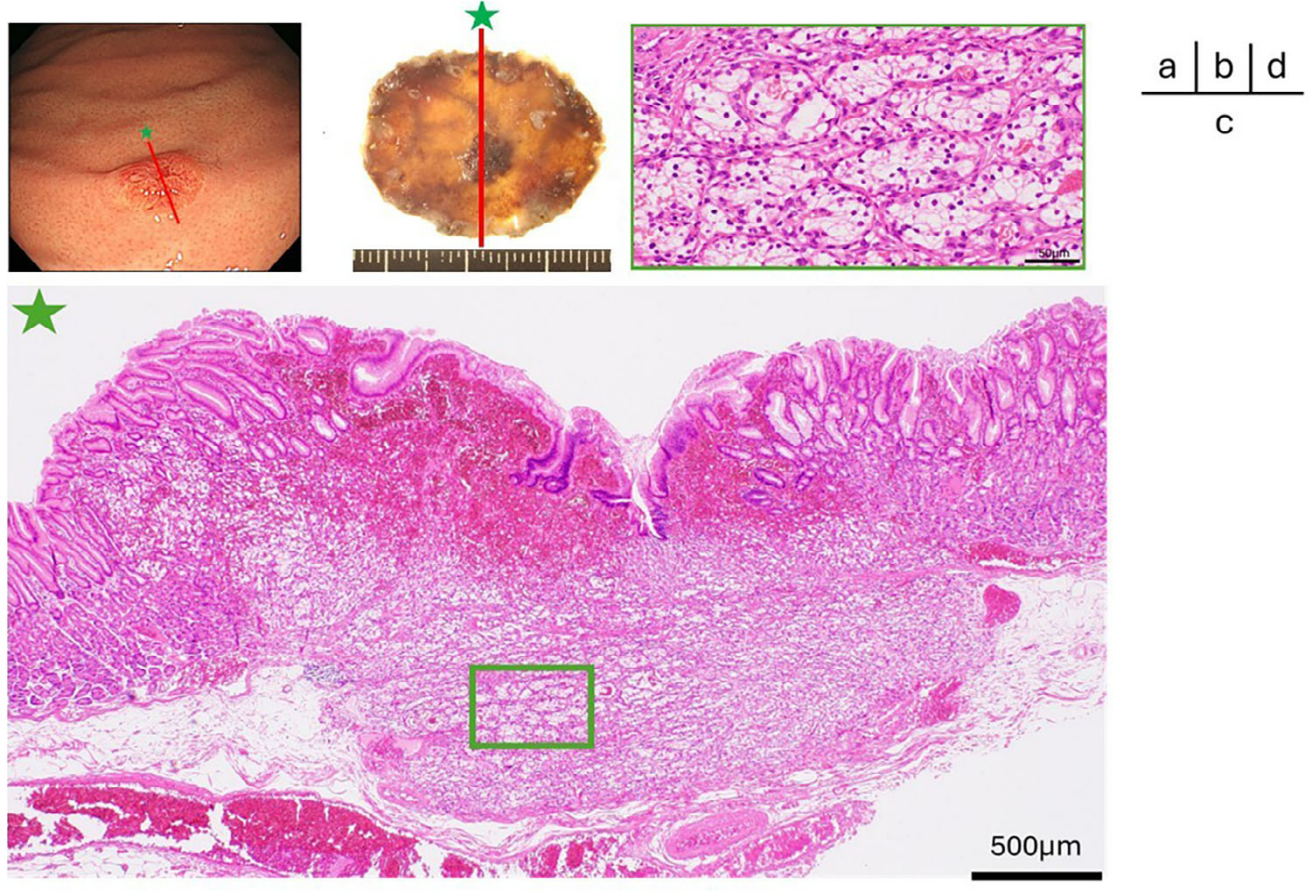
Pathological examination. (a) Endoscopic image with an incision line indicated. (b) Gross specimen. The resection line was placed along the red‐marked area. (c) Hematoxylin and eosin staining. Tumor cell proliferation is observed from the submucosal layer extending partially into the mucosa. The surface mucosal architecture is preserved. (d) Atypical cells with clear and abundant cytoplasm are observed, proliferating in small alveolar nests.

The histopathological findings are shown in Figure 4. The lesion measured 6 × 5 mm. Hematoxylin–eosin staining showed small round nuclei and clear cytoplasm forming small alveolar structures. Cancer cell invasion was observed from the submucosa into parts of the mucosa, with preservation of the surface glandular architecture. No evidence of lymphovascular invasion was observed. The immunohistochemical staining results showed that the cell membranes were positively stained with CD10 and were devoid of reactivity for CK‐7. In PAS with diastase digestion staining, the cell membrane was not stained. These findings distinguished the metastasis of RCC from primary gastric tumors. The final diagnosis was gastric metastasis of a clear cell RCC. The margin of this specimen was pathologically negative.

The patient was followed with regular abdominal CT and EGD. Eight years post‐nephrectomy, a tumor lesion appeared in the pancreatic head. Given that the lesion gradually enlarged, it was considered to be another metastasis of RCC, and treatment with pazopanib hydrochloride was initiated. Thirteen years post‐nephrectomy, there has been no recurrence of the gastric lesion on EGD, and the pancreatic lesion remains under control.

## 3 Discussion

RCC frequently metastasizes to the lungs, liver, brain, and bones at the time of its diagnosis or during the course of treatment. Lyon et al. summarized the anatomical sites of 740 metastasectomies of RCCs and reported that the metastatic sites of RCC are the lungs (36%), bones (13%), nonregional lymph nodes (9%), ipsilateral adrenal gland (8%), contralateral adrenal gland (8%), pancreas (8%), contralateral retroperitoneum (5%), and liver (3%), whereas the gastrointestinal metastasis of RCC is not common. Among gastrointestinal metastases of cancers, cases of gastric metastasis were extremely rare, and an incidence of 0.3%–0.7% was reported in autopsy studies, mainly in lung, breast, melanoma, and esophageal cancers [1].

Gastric metastases of RCC are considered to result from cancer cell hematogenous spread, invading the submucosal layer of the stomach, and often showing submucosal tumor‐like formations in the early stages [2]. They may present various morphologies, ranging from flat lesions, which were similar to our case, to polypoid lesions, depending on the growth pattern. RCC progresses, invading from the submucosa to either the mucosal surface or deeper layers, such as the muscularis propria. When the cancer cells reside in the submucosa, its mucosal surface architecture is often preserved, and the lesion may appear poorly demarcated on EGD. In our case, cancer cells were observed within the submucosa in Endoscopic ultrasonography, and the lesion was covered by a relatively normal‐appearing foveolar epithelium with minimal atypia. We carefully resected for the vertical margin, especially in the center of the lesion. As we should manage bleeding risk given the vascularity of RCC, we carefully coagulated the blood vessels in the submucosa while in ESD. These procedures led to the R0 resection. After ESD, we performed annual EGD and CT every 4–6 months.

In retrospect, a possible recurrence was first noted during routine screening EGD performed at 4 years post‐nephrectomy. Annual EGDs were subsequently performed, and a morphological change noted at 6 years postoperatively prompted biopsy, which confirmed the presence of a gastric metastasis of RCC. In some cases, surgical resections were performed for gastric metastases [3]. In the present case, endoscopic ultrasound findings suggested the tumor was located within the submucosal layer and was considered respectable by endoscopy. Moreover, ESD was performed after obtaining informed consent from the patient. Although a pancreatic tumor was noted at 8 years post‐nephrectomy (2 years after ESD), and its gradual enlargement led to the initiation of systemic chemotherapy at 11 years postoperative year, follow‐up EGD confirmed no evidence of local recurrence at the ESD site on the 13th postoperative year.

Gastric metastases are known to occur as solitary, single, large, ulcerated lesions; few reported cases of metastatic RCC presented as solitary subcentimeter gastric lesions. Some cases of metastatic gastric tumors have been reported after gastrointestinal bleeding, which required curative resection [4]. The most frequent symptoms of gastric metastases include upper gastrointestinal bleeding, anemia, and epigastric pain. The present case is a rare occurrence, as the asymptomatic patient underwent EGD for 2 years to monitor the lesions after a 5‐mm area of erythema was observed. Although some gastric metastases may remain stable over time, such as the present case, there is still a potential risk of gastrointestinal bleeding if the lesions, even subcentimeter lesions, are left untreated for prolonged periods.

The growth rate of renal cancer is basically classified as rapid or slow. Rapid‐growing cases are metastatic lesions identified up to 2 years post‐RCC surgery. However, RCC metastases, particularly well‐differentiated subtypes, can also exhibit a slow‐growing course. Poor prognosis is associated with several factors, including multiple metastases, protruding gastric lesions, and gastric metastases detected within 6.3 years after the therapeutic intervention for renal cancer [5].

Recurrence may occur several years after the surgery, manifesting as pulmonary, hepatic, or cerebral metastases. According to the clinical practice guidelines for RCC, complete surgical resection of pulmonary or pancreatic metastases is associated with improved survival. However, due to the rarity of gastric metastasis of RCC, there are currently no established specific guidelines for its treatment. Dabestani et al. concluded that the complete resection of metastases of RCC is beneficial to the patients’ overall and cancer‐specific survival [6]. In the present case, the treatment for an already systemic disease is controversial for resection or chemotherapy, and the patient also developed pancreatic metastasis 2 years after ESD. However, Table 1 [4, 7, 8, 9, 10] shows that, as subcentimeter lesions also have potential risk for gastrointestinal bleeding, minimally invasive treatment can be acceptable for the metastasis of RCC. If possible, treatments should be less invasive than conventional surgery and systemic chemotherapy. The interval for nephrectomy and gastric metastasis is 2–6 years. In some cases, gastric metastasis was synchronously detected. In two cases, as gastrointestinal bleeding was detected, resection was performed even if there were metastases in other organs.

**TABLE 1:** Clinical data for reported cases of gastric metastasis of renal cell carcinoma.

Author	Year	Age	Gender	Interval for nephrectomy and gastric metastasis (year)	Symptom	Gastrointestinal bleeding	Size (mm)	Tumor location	Gross appearance type	Therapy	Other metastases	Outcome
Kim MY	2012	79	M	synchronous	Yes	No	6	M^b^	Erosive lesion	ESD^c^	None	6 months survival
Sakurai K	2014	61	M	2	Yes	Yes	25	M	Protruded lesion	operation	Brain, lung, and bone	4 months later, dead
Koterazawa S	2020	70	F	synchronous	Yes	No	3	M	Reddish lesion	ESD	None	4 months survival
Chen WG	2022	65	M	5	No	No	11	U^a^	Discoid‐shaped lesion	ESD	Gallbladder and pancreas	1 year survival
Yamashita K	2024	77	M	6	Yes	Yes	15	M	Protruded lesion	EMR^d^	Lung	6 months survival
Present case		62	M	4	No	No	5	M	Reddish polypoid lesion	ESD	None	13 years survival

^a^ U, upper third of the stomach.

^b^ M, middle third of the stomach.

^c^ ESD, endoscopic submucosal dissection.

^d^ EMR, endoscopic mucosal resection.

If curative resection is feasible, an intervention should be considered. In our case, follow‐up EGD confirmed no evidence of local recurrence at the ESD site. In such a case, selecting an appropriate resection method is critical, and ESD may represent a less invasive alternative to surgery. We believe that the accumulation of further cases will be important for establishing evidence‐based management strategies for the treatment of gastric metastasis of RCC.

## Author Contributions

*Conceptualization*: Yuichi Fukami. *Investigation*: Yuichi Fukami, Nagako Nishihira, Kazuki Kawakami, Yuka Hayakawa, Yuki Matsui, and Midori Wakiya. *Supervision*: Masayoshi Fukuda, Hiromichi Shimizu, Kazuo Ohtsuka, and Ryuichi Okamoto. *Writing – original draft*: Yuichi Fukami. *Writing – review & editing*: Yuichi Fukami, Masayoshi Fukuda, Hiromichi Shimizu, Kazuo Ohtsuka, and Ryuichi Okamoto.

## Conflicts of Interest

The authors declare no conflicts of interest.

## Funding

This research did not receive any specific grant from funding agencies in the public, commercial, or not‐for‐profit sectors.

## Data Availability

We declare the availability of our data in our submission.
